# Author Correction: Tissue-resident macrophage survival depends on mitochondrial function regulated by SerpinB2 in chronic inflammation

**DOI:** 10.1038/s41467-026-72166-5

**Published:** 2026-04-17

**Authors:** Sathish Babu Vasamsetti, Samreen Sadaf, Mohammad A. Uddin, Jixing Shen, Ebin Johny, Awishi Mondal, Jonathan Florentin, Liqun Lei, Aleef Mannan, Krithika Sudhakar Rao, John Sembrat, Mauricio Rojas, Ian Sipula, Jake Kastroll, Michael J. Jurczak, Sruti Shiva, Robert M. O’Doherty, Vijay Yechoor, Partha Dutta

**Affiliations:** 1 Pittsburgh Heart, Lung, Blood, and Vascular Medicine Institute, University of Pittsburgh, Pittsburgh, PA USA; 2 Division of Cardiology, Department of Medicine, University of Pittsburgh, Pittsburgh, PA USA; 3 Division of Pulmonary, Critical Care, & Sleep Medicine, Davis Heart & Lung Research Institute, The Ohio State University, Columbus, OH USA; 4 Division of Endocrinology and Metabolism, Department of Medicine, University of Pittsburgh, Pittsburgh, PA USA; 5 Center for Metabolism and Mitochondrial Medicine, University of Pittsburgh, Pittsburgh, PA USA; 6 Department of Pharmacology and Chemical Biology, University of Pittsburgh, Pittsburgh, PA USA; 7 Department of Immunology, University of Pittsburgh, Pittsburgh, PA USA; 8 Department of Bioengineering, Swanson School of Engineering, University of Pittsburgh, Pittsburgh, PA USA; 9 The Center for Cardiovascular Inflammation, University of Pittsburgh, Pittsburgh, PA USA; 10 Veterans Affairs Pittsburgh Healthcare System, Pittsburgh, PA USA

**Keywords:** Phagocytes, Type 2 diabetes

Correction to: *Nature Communications* 10.1038/s41467-026-69196-4, published online 12 February 2026

In the version of this article initially published, there was a presentation error where the VAT p-Akt blot shown in the third lane of Fig. 2d was incorrect due to a figure preparation mistake. The figure and source data have been updated in the HTML and PDF versions of the article. For comparison, the original figure is available as Supplementary Information accompanying this amendment.

## Supplementary information

Original, uncorrected Fig. 2

