# Supplementary material for: Tissue-resident macrophage survival depends on mitochondrial function regulated by SerpinB2 in chronic inflammation
Source: Nat Commun. 2026 Feb 12;17:1493. doi: 10.1038/s41467-026-69196-4 (PMC12902017; doi:10.1038/s41467-026-69196-4)
Supplement: Supplementary file 1 — Supplementary Information [file 41467_2026_69196_MOESM1_ESM.pdf]

Tissue-resident macrophage survival depends on mitochondrial function regulated by SerpinB2 in chronic inflammation

Sathish Babu Vasamsetti[1,2†], Samreen Sadaf[1†], Mohammad A Uddin[1], Jixing Shen[1], Ebin Johny[1], Awishi Mondal[1], Jonathan Florentin[1], Liqun Lei[1], Aleef Mannan[1], Krithika Sudhakar Rao[1], John Sembrat[1], Mauricio Rojas[3], Ian Sipula[4], Jake Kastroll[4], Michael J Jurczak[4,5], Sruti Shiva[1,6], Robert M. O'Doherty[1,4], Vijay Yechoor[1,4], Partha Dutta[1,2,7,8,9,10]*

[1]Pittsburgh Heart, Lung, Blood, and Vascular Medicine Institute, University of Pittsburgh, Pittsburgh, PA, USA
[2]Division of Cardiology, Department of Medicine, University of Pittsburgh, Pittsburgh, PA, USA
[3]Division of Pulmonary, Critical Care, & Sleep Medicine, Davis Heart & Lung Research Institute, The Ohio State University, Columbus, OH, USA
[4]Division of Endocrinology and Metabolism, Department of Medicine, University of Pittsburgh, Pittsburgh, PA, USA
[5]Center for Metabolism and Mitochondrial Medicine, University of Pittsburgh, Pittsburgh, Pennsylvania, USA
[6]Department of Pharmacology and Chemical Biology, University of Pittsburgh, Pittsburgh, PA, USA
[7]Department of Immunology, University of Pittsburgh, Pittsburgh, PA, USA
[8]Department of Bioengineering, Swanson School of Engineering, University of Pittsburgh, Pittsburgh, PA, USA
[9]The Center for Cardiovascular Inflammation, University of Pittsburgh, Pittsburgh, PA, USA
[10]Veterans Affairs Pittsburgh Healthcare System, Pittsburgh, PA 15240, USA

[†]Contributed equally to this work.

*Corresponding author:
Partha Dutta, DVM, PhD. FAHA
Center for Pulmonary Vascular Biology and Medicine
Pittsburgh Heart, Lung, Blood, and Vascular Medicine Institute,
Division of Cardiology, Department of Medicine
University of Pittsburgh School of Medicine
200 Lothrop Street BST1720.1
Pittsburgh, PA USA 15213
Tel: 412-383-7277
Email: duttapa@pitt.edu

Conflict of interest: The authors have declared that no conflict of interest exists.

SUPPLEMENTAL INFORMATION:

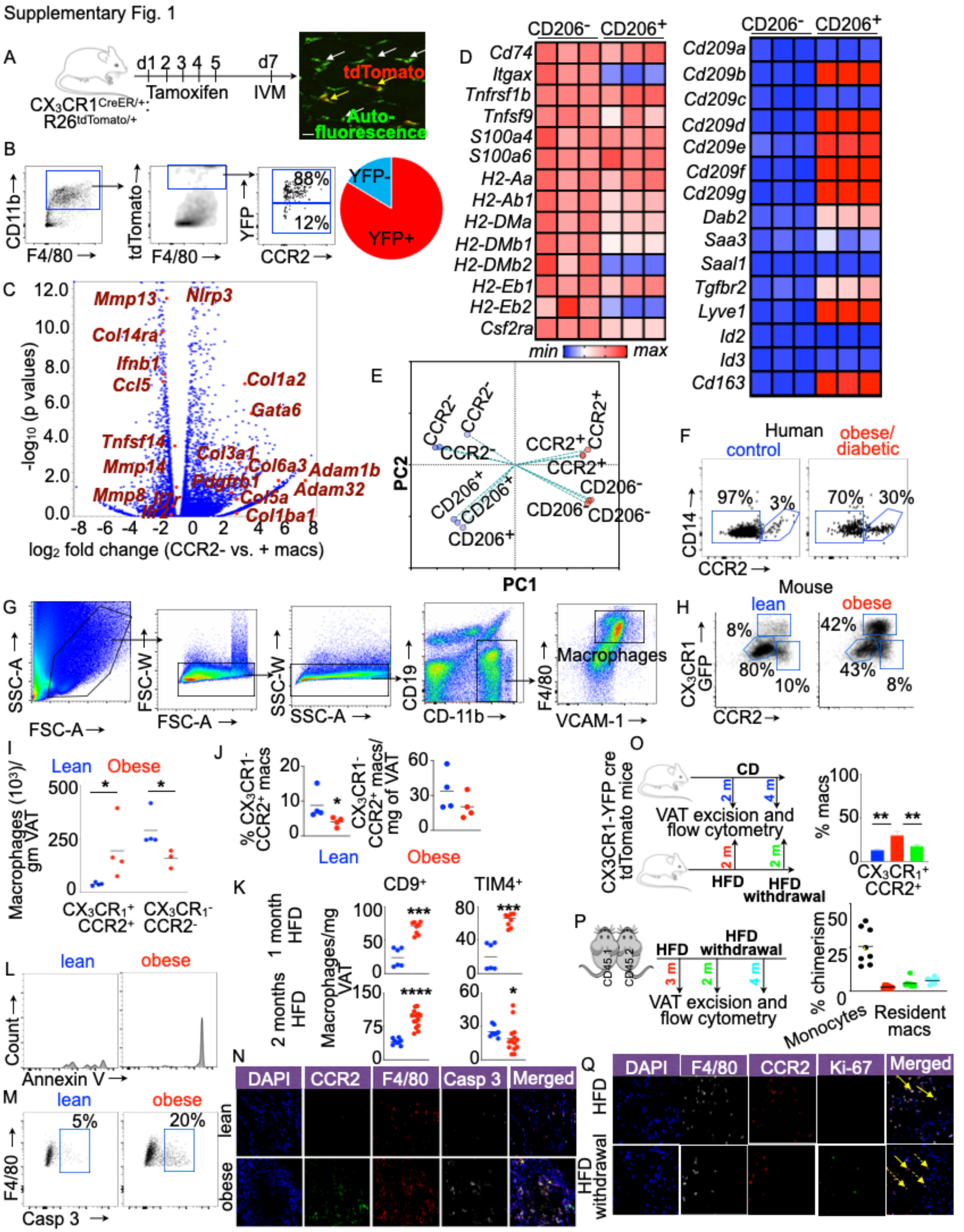

Supplementary Fig. 1: Monocyte-derived macrophages and resident macrophages of VAT

express different transcriptomic profiles, and the resident macrophage population decreases in obesity. A) Genetic fate mapping in $CX_3CR_1^{CreER/+}$ $ROSA^{tdTomato/+}$ mice to identify the origin of CX_3CR1^+ $CCR2^+$ ($tdTomato^+$) macrophages. The yellow and white arrows indicate $tdTomato^+$ and $tdTomato^-$ macrophages, respectively. Scale bar = 10 µm. B) $CX3CR1^{CreER/+}$ $ROSA^{tdTomato/+}$ mice were injected with tamoxifen, and the flow cytometry analysis was performed four weeks later (n=3/ group). C) Volcano plot showing the differentially expressed genes between the cell populations. D) The heat map of the genes affecting insulin sensitivity and inflammation in adipose $CD206^-$ vs. $CD206^+$ macrophages of lean mice (n=3/group). E) The PCA plot depicts the transcriptomic relations between the macrophage subsets. F-H) The flow cytometric plots show monocyte-derived and VAT resident macrophage frequencies in lean and obese patients (F) and mice (G&H). I) The numbers of CX_3CR1^+ $CCR2^+$ and CX_3CR1^- $CCR2^-$ VAT macrophages in obese mice (four months of HFD) were enumerated by flow cytometry (n=4/group). J) The frequencies and numbers of CX_3CR1^- $CCR2^+$ macrophages were assessed by flow cytometry (n=4/group). K) $CD9^+$ and $TIM4^+$ VAT macrophages at one and two months after HFD feeding were enumerated by flow cytometry (n=6-10/group). L and M) The flow cytometric plots showing annexin V (L) and caspase 3 (M) staining in adipose macrophages. N) The confocal images show CCR2, F4/80, and caspase 3 staining in VAT of lean and obese mice. The dashed arrows indicate Caspase 3^+ $CCR2^-$ macrophages. Scale bar = 30 µm. O) Experimental design and quantification of the VAT macrophage subsets in lean and obese $CX_3CR1^{creER/+}$ $ROSA^{tdTomato}$ mice before and after withdrawal of HFD (n=7/ group). P) Experimental design to decipher the origin of VAT resident macrophages after HFD withdrawal. Chimerism in monocytes and VAT resident macrophages was quantified using flow cytometry (n=8/group). Q) Confocal images of Ki-67^+ VAT resident macrophages after HFD withdrawal. The solid and dashed arrows indicate $CCR2^+$ and $CCR2^-$ macrophages, respectively. Scale bar = 30 µm. Mean ± s.e.m. * $P < 0.05$, ** $P < 0.01$, *** $P < 0.001$, **** $P < 0.0001$. The Mann Whitney test (two-tailed) was used to determine the significance between two groups.

Supplementary Fig. 2

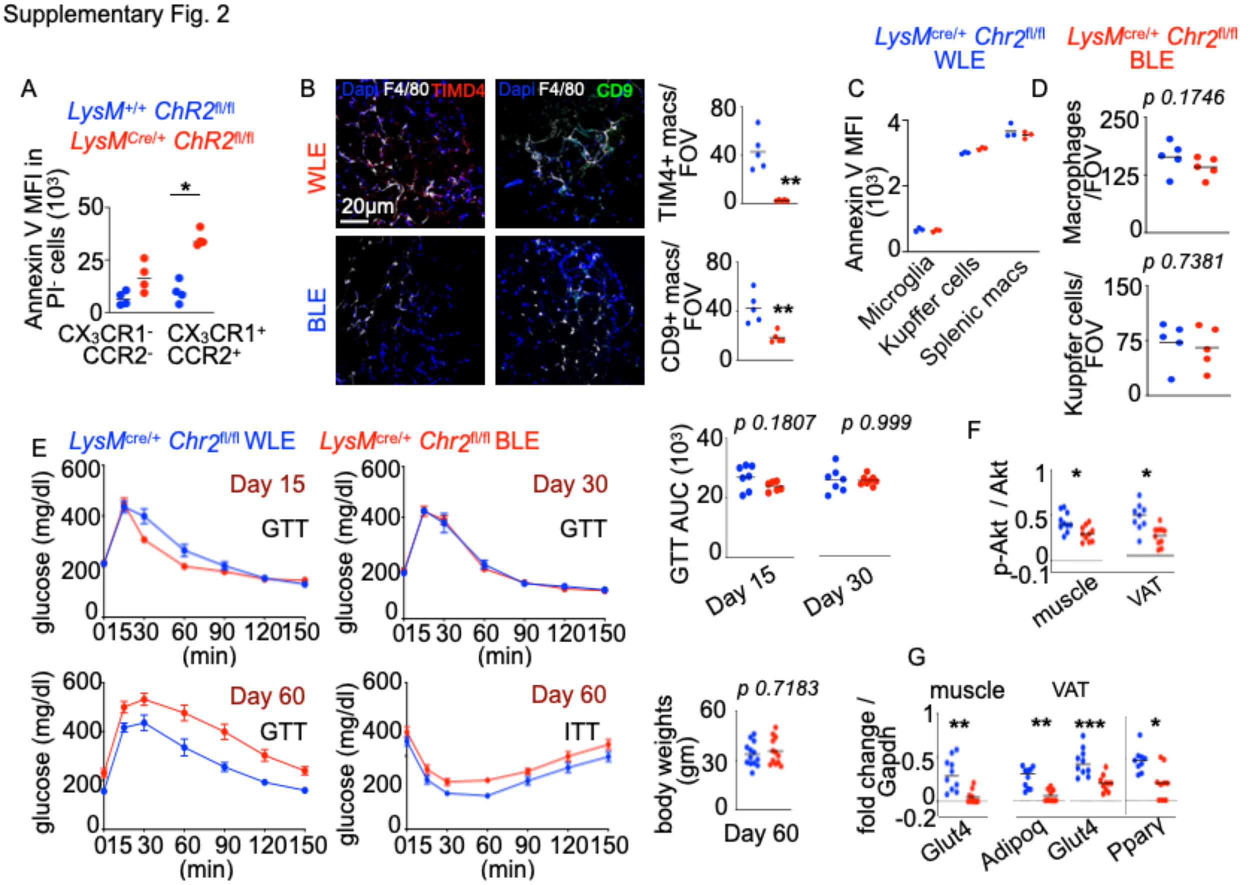

Supplementary Fig. 2: Loss of VAT resident macrophages exacerbates obesity-induced metabolic complications. A) The frequencies of apoptotic macrophages after blue light exposure in *LysM*$^{+/+}$ *ChR2*$^{fl/fl}$ and *LysM*$^{cre/+}$ *ChR2*$^{fl/fl}$ mice were calculated by flow cytometry (n=4/ group). B) VAT of *LysM*$^{cre/+}$ *ChR2*$^{fl/fl}$ mice was exposed to either white light (WLE) or blue light (BLE). The numbers of TIM4^{+} and CD9^{+} macrophages were enumerated by confocal microscopy (n=5/group). Scale bar= 20 µm. C) Annexin V mean fluorescence intensities were calculated by flow cytometry in microglia, Kupffer cells, and splenic macrophages in *LysM*$^{cre/+}$ *Chr2*$^{fl/fl}$ mice after white and blue light exposure to VAT (n=3/group). D) Hepatic macrophages and Kupffer cells were enumerated by confocal microscopy in these mice (n=5/group). E) Glucose tolerance test (GTT) (n=7-12/ group), insulin tolerance test (ITT) (n=7-12/ group), and bodyweights (A) (n=5-13/group), and quantifications of the indicated proteins by immunoblot (F and G) in blue or white light-exposed obese *LysM*$^{cre/+}$/*Chr2*$^{fl/fl}$ mice were performed (n=8-10/group). Mean ± s.e.m. * $P < 0.05$, ** $P < 0.01$, *** $P < 0.001$. The Mann Whitney test (two-tailed) was used to determine the significance between two groups.

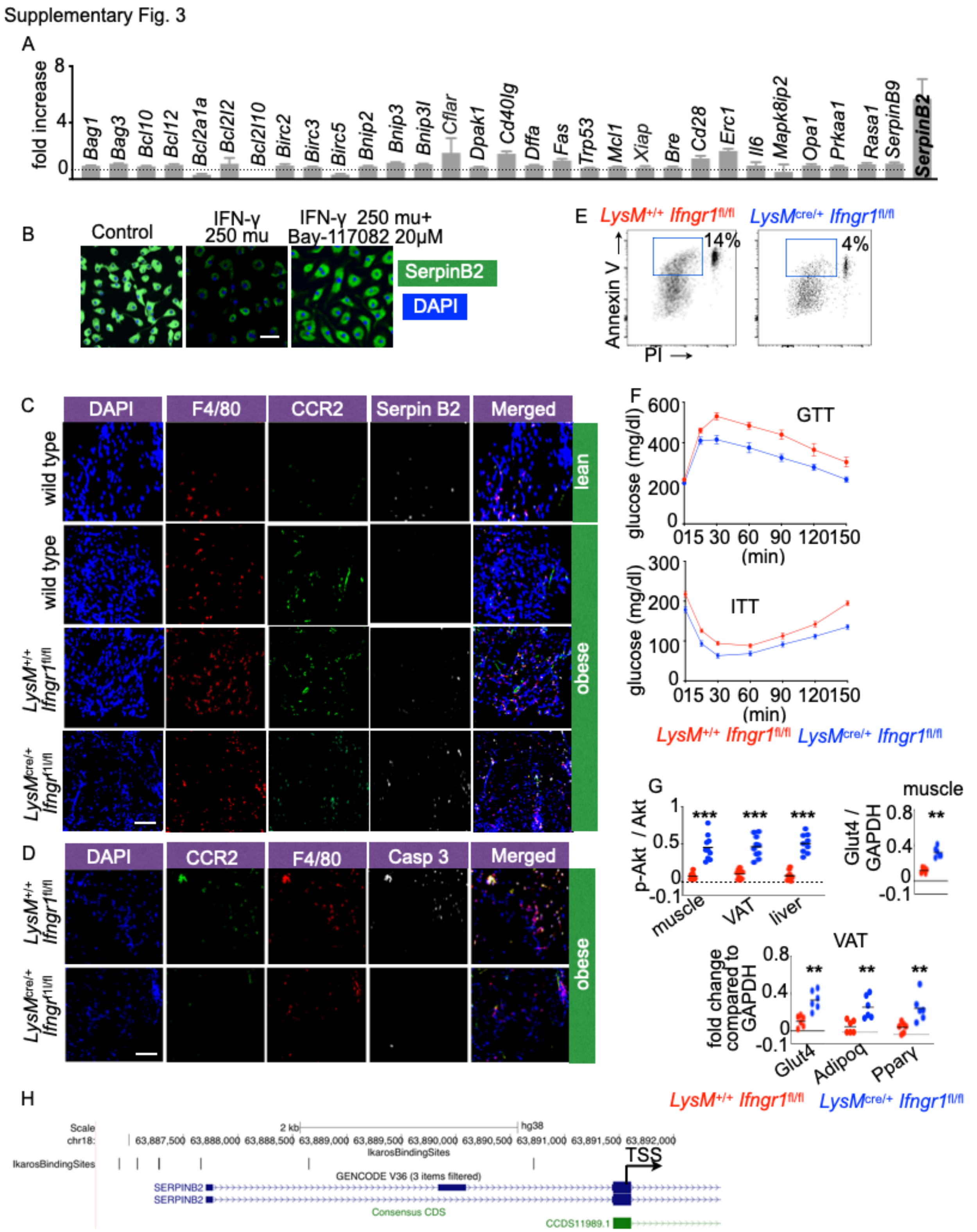

Supplementary Fig. 3: IFN-γ-mediated SerpinB2 downregulation leads to apoptosis of VAT resident macrophages in obesity. A) The bar graph displays the levels of the cell survival regulating genes in CX3CR1$^-$ CCR2$^-$ VAT resident macrophages compared to CX3CR1$^+$ CCR2$^+$ VAT monocyte-derived macrophages using RNA sequencing (n=3/group). B) SerpinB2 staining

in PMA-differentiated THP-1 macrophages treated with IFN-γ in the presence or absence of NF-kB inhibitor Bay-118072. Scale bar = 10 μm. C and D) Confocal images showing SerpinB2 (C) and caspase 3 (D) expression in VAT macrophages of lean and obese wildtype, and obese *LysM* $^{+/+}$ *Ifngr1*$^{fl/fl}$ and *LysM*$^{cre/+}$ *Ifngr1*$^{fl/fl}$ mice. Scale bar = 30 μm. E) Representative flow cytometric plots showing annexin V^{+} PI^{-} apoptotic cells. F and G) GTT and ITT (n=10-11/ group) (F) and quantifications of the proteins using immunoblot (n=5-9/ group) (G) in obese *LysM* $^{+/+}$ *Ifngr1*$^{fl/fl}$ and *LysM*$^{cre/+}$ *Ifngr1*$^{fl/fl}$ mice. H) Schematic generated using the UCSC Genome Browser to show the Ikaros binding sites on the human *SerpinB2* promoter. Mean ± s.e.m. * $P < 0.05$, ** $P < 0.01$, ***$P < 0.001$. The Mann Whitney test (two-tailed) was used to determine the significance between two groups.

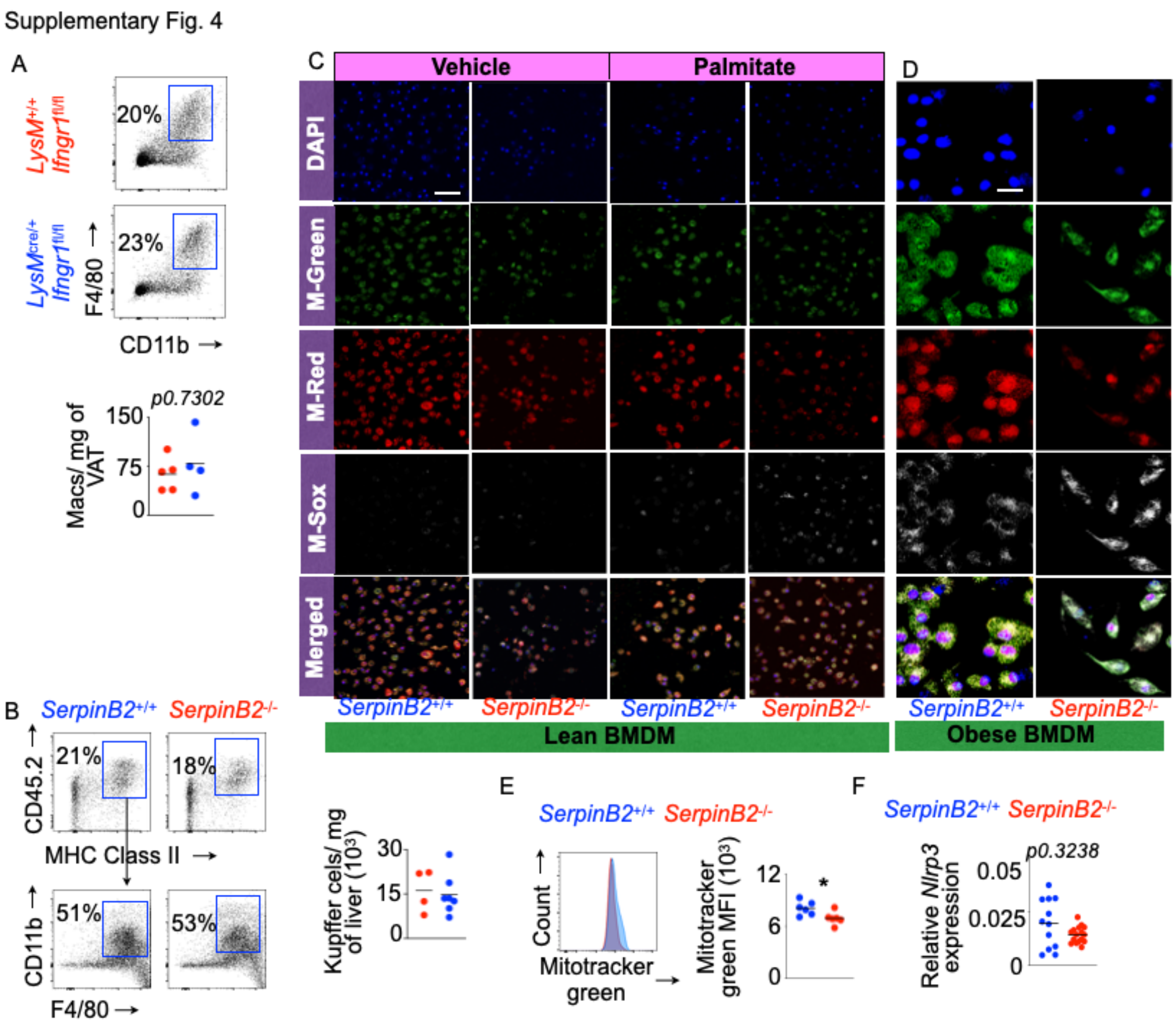

Supplementary Fig. 4: Mitochondrial ROS mediates cytochrome c translocation from the mitochondria to cytoplasm. A) *LysM*$^{+/+}$ *Ifngr1*$^{fl/fl}$ and *LysM*$^{Cre/+}$ *Ifngr1*$^{fl/fl}$ mice were fed with an HFD and macrophages were analyzed by flow cytometry (n=4-5/group). B) Kupffer cells in *SerpinB2*$^{+/+}$ and *SerpinB2*$^{-/-}$ mice were evaluated by flow cytometry (n=4-7/group). C and D) Confocal images of mitotracker in *SerpinB2*$^{+/+}$ and *SerpinB2*$^{-/-}$ BMDM cultured in the presence or absence of palmitate (200 µM) and mitotempol (250 µM). The cells were stained with mitotracker green (200 nM), mitotracker red (200 nM), and mitosox (5 µM). The BMDM were obtained from either lean (C) or obese (D) mice. scale bar= 5 µm (C), 20 µm (D). E) BMDM isolated from *SerpinB2*$^{+/+}$ and *SerpinB2*$^{-/-}$ mice were stained with mitotracker green and analyzed by flow cytometry (n=5-6/group). F) The expression of *Nlrp3* was measured by qPCR (n=12=16/ group). Mean ± s.e.m. * $P < 0.05$. The Mann Whitney test (two-tailed) was used to determine the significance between two groups.

Supplementary Fig. 5

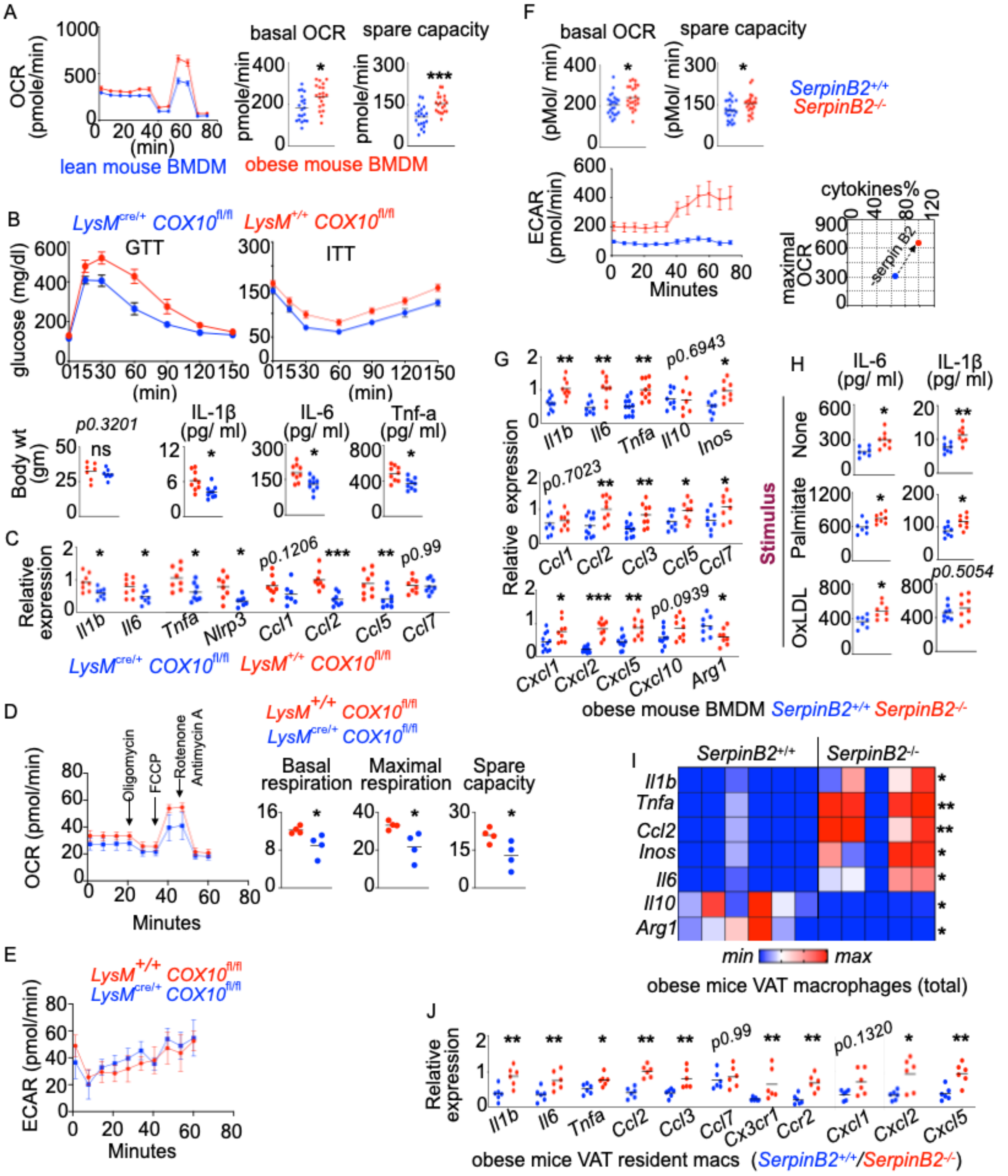

Supplementary Fig. 5: *SerpinB2* deficiency exacerbates inflammation. A) OCR, basal OCR, and spare capacity were calculated using the SeaHorse assay (n=20/group). B and C) *LysM*$^{+/+}$ *COX10*$^{fl/fl}$ and *LysM*$^{cre/+}$ *COX10*$^{fl/fl}$ mice were fed with an HFD for four months. GTT and ITT were performed, and bodyweights were measured (n=8-9/ group, combined data of two independent experiments). The inflammatory cytokines were quantified by ELISA in BMDM culture supernatants (B) and by qPCR in BMDM (C) (n=8-9/group). D-E) BMDM was collected from

LysM$^{+/+}$ *COX10*$^{fl/fl}$ and *LysM*$^{Cre/+}$ *COX10*$^{fl/fl}$ mice and the SeaHorse assay was performed to measure oxygen consumption rate (D) and extra-cellular acidification rate (E) in these cells (n=4/group). F-J) *SerpinB2*$^{+/+}$ and *SerpinB2*$^{-/-}$ mice were fed with an HFD for four months. F) OCR, basal OCR, and spare capacity were examined by SeaHorse analysis (n=24/group). The correlation between maximal OCR and cytokine levels is shown. The levels of the inflammatory cytokines were ascertained in BMDM by qPCR (G) (n=8-11/group), BMDM supernatant by ELISA (H) (n=7-8/group), and in total VAT macrophages (I) (n=5-6/group), and VAT resident macrophages (J) (n=6/group) isolated from obese mice by qPCR. Mean ± s.e.m. * $P < 0.05$, ** $P < 0.01$, ***$P < 0.001$, ****$P < 0.0001$. The Mann Whitney test (two-tailed) was used to determine the significance between two groups.

Supplementary Fig. 6

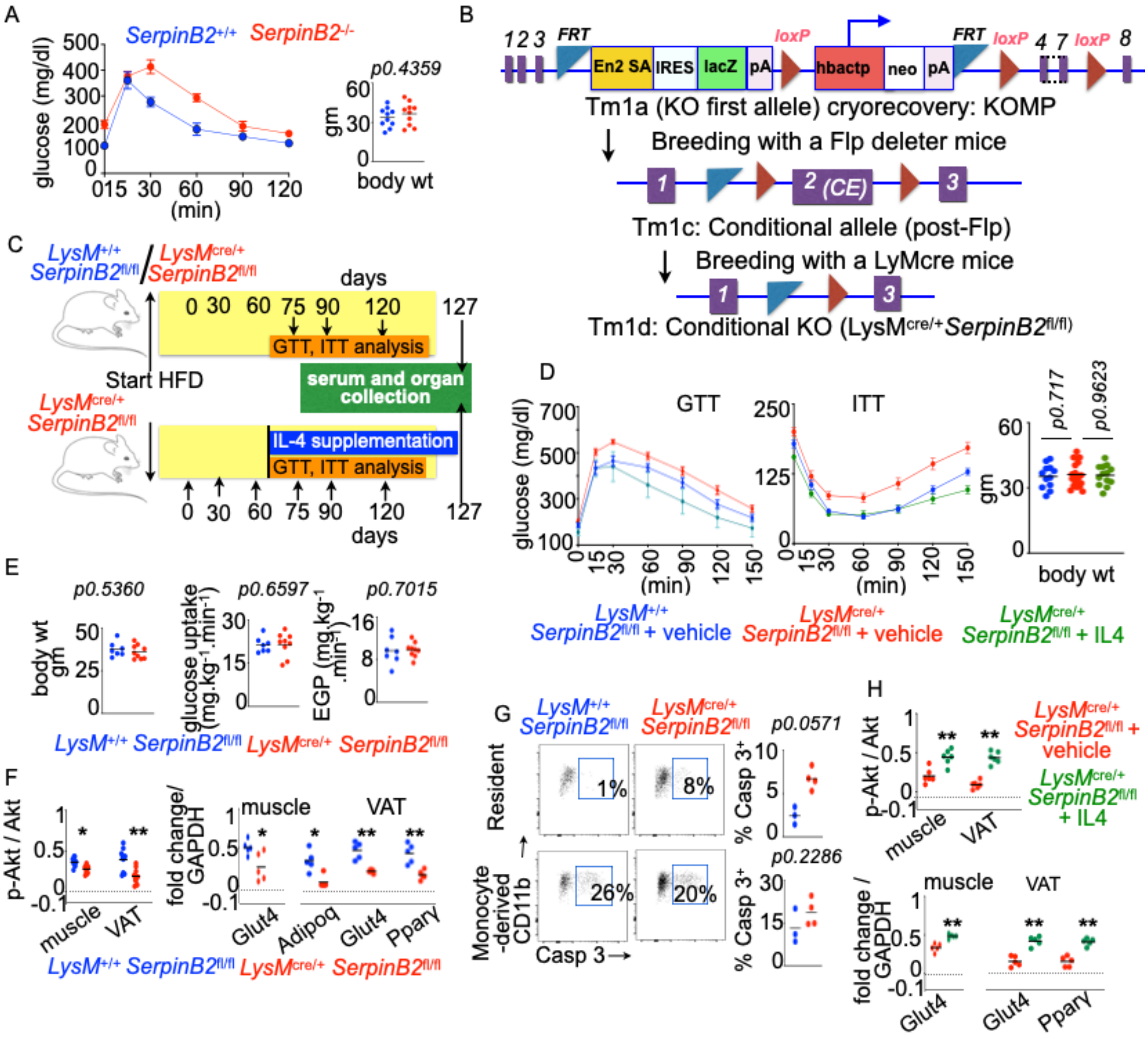

Supplementary Fig. 6: IL-4 treatment prevents glucose intolerance in the absence of SerpinB2 in diet-induced obesity. A) A GTT in obese *SerpinB2*$^{+/+}$ and *SerpinB2*$^{-/-}$ mice was performed, and body weights were measured (n=10-12/ group, combined data of at least three independent experiments). B-G) *LysM*$^{+/+}$ *SerpinB2*$^{fl/fl}$ and *LysM*$^{cre/+}$ *SerpinB2*$^{fl/fl}$ mice were fed with an HFD for four months. A group of *LysM*$^{cre/+}$ *SerpinB2*$^{fl/fl}$ mice was supplemented with IL-4. B) Schematic showing the steps of *LysM*$^{cre/+}$ *SerpinB2*$^{fl/fl}$ mouse generation. C) Schematic diagram of the experimental setup. GTT, ITT, and bodyweights (D) (n=12-16/ group, combined data of at least two independent experiments) were measured. Body weight, steady state whole-body glucose uptake, and endogenous (hepatic) glucose production were calculated using a hyperinsulinemic-euglycemic clamp study (E) (n=7-9/group). The quantification of the proteins by immunoblot (F and H) (n=5-8/group), and the assessment of apoptotic VAT macrophages by flow cytometry (G) were carried out. (n=3-4/group). Mean ± s.e.m. *P<0.05, **P<0.01. The Mann Whitney test (two-tailed) was used to determine the significance between two groups.

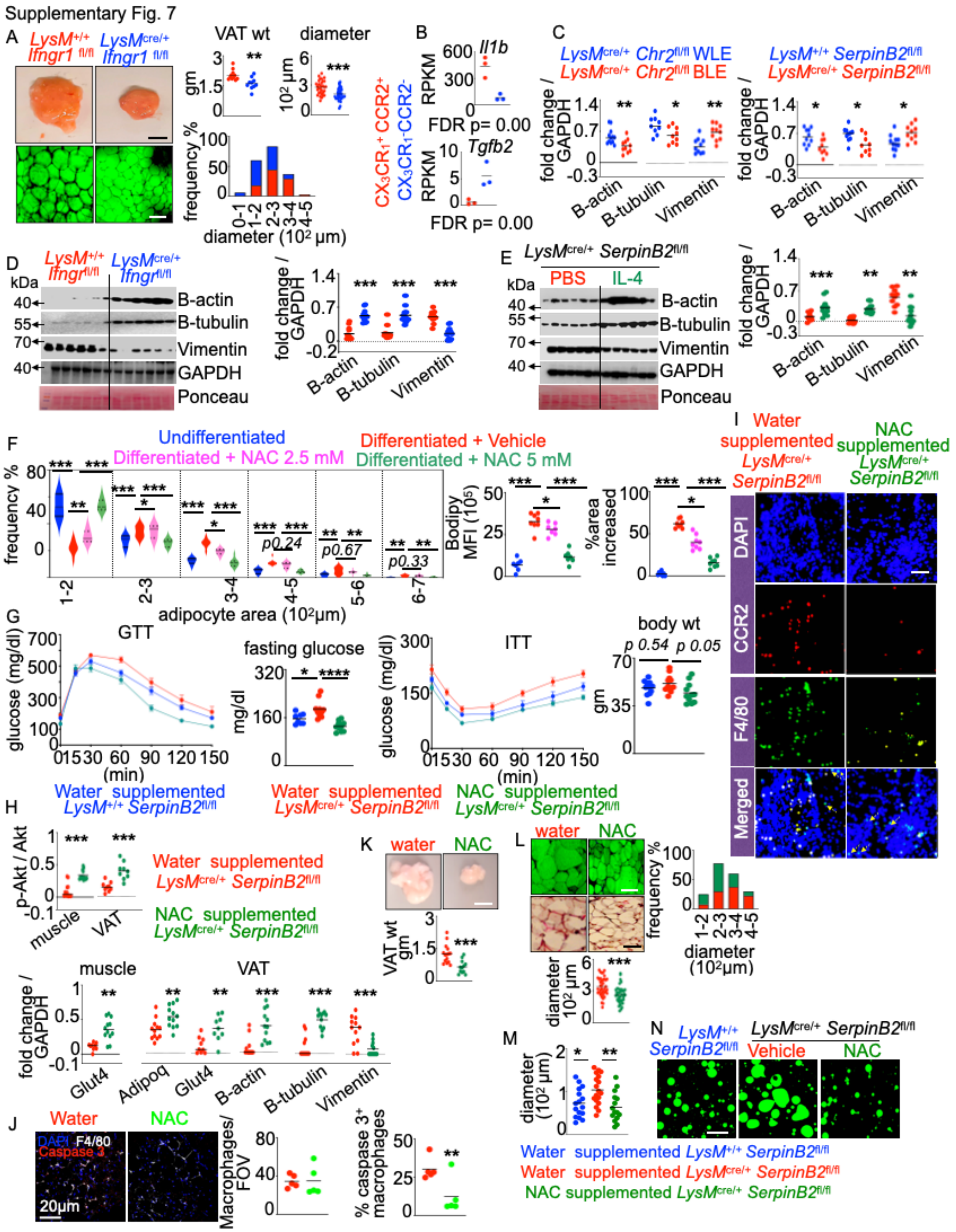

Supplementary Fig. 7: Glutathione (GSH) prevents adipocyte hypertrophy and VAT expansion in diet-induced obesity. A) VAT weights and adipocyte diameters by BODIPY staining were assessed in obese *LysM* $^{+/+}$ *Ifngr1*$^{fl/fl}$ and *LysM*$^{cre/+}$ *Ifngr1*$^{fl/fl}$ mice (n=10-14/group).

Scale bar = 20 µm for the BODIPY and 200µm for VAT images. B) RNA sequencing was performed in monocyte-derived and resident macrophages sorted from VAT. The RPKM values for *Il1b* and *Tgfb2* are plotted (n=3/ group). C-E) Quantifications of the proteins by immunoblot in obese *LysM*$^{cre/+}$ *Chr2*$^{fl/fl}$ mice exposed to either white (WLE) or blue (BLE) light and obese *LysM*$^{+/+}$ *SerpinB2*$^{fl/fl}$ and *LysM*$^{cre/+}$ *SerpinB2*$^{fl/fl}$ mice (n=8-12/group) (C), *LysM* $^{+/+}$ *Ifngr1*$^{fl/fl}$ and *LysM*$^{cre/+}$ *Ifngr1*$^{fl/fl}$ mice (n=8-12/group) (D), and *LysM*$^{cre/+}$ *SerpinB2*$^{fl/fl}$ mice treated with either vehicle or IL-4 (n=8-12/group) (E). F) Quantification of 3T3 L1 adipocyte area, BODIPY MFI, and frequency of adipocytes (n=10-12/group). BODIPY MFI and the area of adipocytes were quantified by ImageStreamX Mark II in 3T3 L1 differentiated adipocytes in the presence of NAC (n=10-12/group), Scale bar = 5 µm. G, H, and I) Obese *LysM*$^{cre/+}$ *SerpinB2*$^{fl/fl}$ and *LysM*$^{cre/+}$ *SerpinB2*$^{fl/fl}$ mice treated with vehicle or NAC. G) GTT and ITT were performed, and fasting glucose concentration in the serum and body weights were measured (n=10-16/group, combined data of at least two independent experiments). H) The indicated proteins were measured by immunoblot (n=10-12/group). I) Confocal images showing CCR2^{-} macrophages (dashed arrows) in VAT. Scale bar = 30 µm. J) Total and apoptotic VAT macrophages were determined by confocal microscopy (n=5/group). VAT weights were determined (K), and adipocyte diameter (L) was quantified by BODIPY staining. (n=8-10/group). Scale bar=20 µm for the BODIPY and the bright filed images, and 200 µm for VAT images. M-N) Average adipocyte diameters were calculated in 3T3-L1-derived adipocytes cultured in the presence of conditioned medium of BMDM of obese *LysM*$^{cre/+}$ *SerpinB2*$^{fl/fl}$ and *LysM*$^{cre/+}$ *SerpinB2*$^{fl/fl}$ mice supplemented with either water or NAC. Scale bar = 20 µm. Mean ± s.e.m. * $P < 0.05$, ** $P < 0.01$, ***$P < 0.001$, ****$P < 0.0001$. The Mann Whitney test (two-tailed) was used to determine the significance between two groups.

Patient	Age range	Sex	BMI	Diabetic
HAC-2	48-51	F	29.737	No
HAC-4	50-53	M	27.31	No
HAC-6	89-92	M	28.1	Yes
HAC-10	19-23	F	20.91	No
HAC-12	22-26	F	25.71	Yes
HAC-13	57-60	F	33.30	Yes
HAC-15	55-58	F	28.125	No
HAC-17	18-21	M	22.53	No
HAC-18	63-67	M	29.04	No
HAC-19	83-86	F	22.32	No
HAC-20	71-74	M	34.6	Yes
HAC-22	41-43	F	45.54	Yes
HAC-26	57-60	M	28	No
HAC-28	17-20	F	20.5	No
HAC-30	81-85	M	22.4	No
HAC-31	83-86	F	20.05	No
HAC-32	62-67	F	41.03	Yes
HAC-33	75-78	M	25.6	Yes
HAC-38	55-58	M	39	No

Supplementary Table 1: The demographic information of patients. Mesenteric adipose tissues from deceased donors were collected. The demographic information of these patients is provided in this table. The age ranges instead of actual ages have been provided to avoid indirect identification of the patients.

**Reagent or resource**	**Source**	**Identifier**
**Chemicals**		
60% high fat diet	Research Diets Inc	D12492
Annexin V staining kit	BD Biosciences	556547
Bay-117082	MedChemExpress	HY-13453
BODIPY	ThermoFisher Scientific	D3922
Bovine serum albumin	Fisher Scientifics	BP1600-1
Phorbol-12-myristate-13-acetate	Calbiochem	524400
Camptothecin	Sigma-Aldrich	C9911
Caspase 3 staining kit	BD Biosciences	550914
cDNA synthesis kit	Applied Biosystems	4387406
Collagenase-1	Sigma-Aldrich	C00130
Collagenase -IV	Worthington	LS004209
Collagenase-IX	Sigma-Aldrich	C7657
Cycloheximide	Alfa Aesar	J6690103
Cytosol/mitochondria extraction kit	Biovision	K25625
DAPI	Invitrogen	D1306
Dream Taq PCR Master mix [65]	ThermoFisher Scientific	K1071
Fixation buffer for nuclear staining	BD biosciences	554655
Free fatty acid assay kit	Bioassay Systems	EFFA-100
Free glycerol assay kit	Bioassay Systems	EGLY-200
Glucose strips	Fisher Scientific	23111276
Hyalouronidase	Sigma-Aldrich	H35006
Mouse IL-4 recombinant	R&D	404-ML-050/CF
Immuno-blot HRP substrate	Millipore	WBLUR0500
Insulin	Fisher Scientifics	12-585-014
Insulin ELISA kit	Mercordia Diagnostics	10-1247-01
IFN-γ	Invitrogen	HC4031
L-Glutamine	Gibco	25030-081
Laemmli SDS sample buffer, reducing	Alfa Aesar	J61337
Lenalidomide	MedChemExpress	HYA0003
Mitotempol	Fisher Scientifics	NC1229394
Mitotracker green	Invitrogen	M7514
Mitotracker deep red	Invitrogen	M22426

Mitosox red	Invitrogen	M36008
N-acetyl cysteine	Research Products	A10040
Palmitate	Acros Organics	416700050
Penicillin-streptomycin mixture	Fisher Scientifics	ICN1670249
Permeabilization buffer for nuclear staining	BD Biosciences	558050
Picopure TM RNA isolation kit	Applied Biosystems	12204-01
Phosphatase inhibitor cocktail	Roche	04906845001
Propidium iodide	BioLegend	421301
Protease inhibitor cocktail	Pierce	88666
Proximity ligation assay kit	Sigma-Aldrich	DUO92004
RBC lysis buffer	BioLegend	420302
RIPA buffer	R&D	J63324
RNA later	Sigma-Aldrich	R0901
RNeasy Mini kit	Qiagen	74104
RPMI 1640 medium	HyClone	SH30027LS
SYBR green PCR master mix	Applied Biosystems	A25776
TNF	R&D	410-MT
Triglycerides liquid reagent	Pointe Scientific	T7532120
Vector shield DAPI	Vector Laboratories	H1200
**Flowcytometry Antibodies**		
Human CCR2	BioLegend	357206
Human CD11C	BD Biosciences	563404
Human CD14	BD Biosciences	555399
Human CD16	BD Biosciences	560195
Human CD206	BD Biosciences	564063
Human CD24	BD Biosciences	561647
Human CD45	BD Biosciences	564585
Human HLA-DR	BD Biosciences	565127
Mouse CD 115	eBioscience	46-1152-82
Mouse CD11b-Biotin	BioLegend	101204
Mouse CD11b	BD Biosciences	557657
Mouse CD11c-Biotin	BioLegend	117304
Mouse CD11c	BioLegend	117338
Mouse CD45.1	BioLegend	110730

Mouse CD45.2	BioLegend, BD Biosciences	109820, 560693
Mouse CD64	BD Biosciences	558455
Mouse F4/80	BioLegend	123114
Mouse Ly-6C	BioLegend	128006
Mouse Ly-6G	BD Biosciences	563979
Mouse MHC-II	BioLegend	107620
Streptavidin BV605, BV510	BD Biosciences	563260, 563261
**Cell lines**		
THP-1 cells	ATCC	TIB-202
3T3-L1	ATCC	CL-173
**Mice**		
Cx3cr1$^{\text{cre ER}}$	JAX	021160
Cx3cr1$^{\text{GFP/GFP}}$	JAX	005582
CD45.1	JAX	002014
C57BL/6 (CD45.2)	JAX	000664
Cox10$^{\text{fl/fl}}$	JAX	024697
Flp recombinase expressing	JAX	009086
Ifngr$^{\text{fl/fl}}$	JAX	025394
LysM$^{\text{cre/cre}}$	JAX	004781
Serpin B2$^{-/-}$	JAX	007234
Serpin B2 Tm1c	KOMP	CSD66707
ROSA-tdTomato	JAX	007914
**Immunofluorescence and Immunoblot antibodies**		
Adiponectin	CST	2789
Akt	CST	4691
p-Akt ser 473	CST	4060
Beta- actin	CST	4970
Beta- tubulin	CST	2128
CCR2	Bio-Rad	AAM72
CD 11b	Abcam	Ab133357
CD 68	eBioscience<br>R&D systems	14-0681-82,<br>ab125212
Cleaved caspase 3	Abcam	Ab13847
CX_3CR1	Abcam	Ab8021
Cytochrome C	Novus Biologicals	SC6908
F4/80	Invitrogen	MA1-91124
GAPDH	CST	2118
Glut4	Fisher Scientifics	MA183191
IgG	CST	2729

Ki67	CST	9129
PPAR-gamma	CST	2435
Serpin B2	Invitrogen, R&D	PA5-27857, MAB855
Vimentin	Sigma	SAB4200716
Software and algorithms		
Prism 7 and 8	GraphPad	
Fiji (Image J)		

Supplementary Table 2: Sources of the reagents, cell lines, and mice used for the study. The reagents/resources, and their sources and identifiers are included in this table.

Gene name	Primer Sequence
Abca1-F (M)	GGAGCCTTTGTGGAACTCTTCC
Abca1-R (M)	CGCTCTCTTCAGCCACTTTGAG
Abca4-F (M)	CGCTCTCTTCAGCCACTTTGAG
Abca4-R (M)	TCCAGCATCCTCTGTGACCTTC
Abcg1-F (M)	TCCAGCATCCTCTGTGACCTTC
Abcg1-R (M)	GCATGATGCTGAGGAAGGTCCT
Abcg3-F (M)	CCTTTCTGCCATCAGCTCAAGTG
Abcg3-R (M)	TTAGGACACGGAAAGCAGTGCC
Abcg4-F (M)	TTCAAGGGCGTGGTTACCAACC
Abcg4-R (M)	GGTTCAGGTCTCCATACTCTCC
Adipoq-F(M)	TGTTCCTCTTAATCCTGCCCA
Adipoq-R (M)	CCAACCTGCACAAGTTCCCTT
Adpn-F (M)	TGT TCC TCT TAA TCC TGC CCA
Adpn-R (M)	CCA ACC TGC ACA AGT TCC CTT
Arg-1-F (H)	ACA GTT TGG CAA TTG GAA GCA
Arg-1-R (H)	CAC CCA GAT GAC TCC AAG ATC AG
Bmp3-F (M)	ACTCCGTGAGACTGAGCCAA
Bmp3-R (M)	CCTGTCATAGAGCCACAGCATA
Cav1-F (M)	ATGTCTGGGGGCAAATACGTG
Cav1-R (M)	CGCGTCATACACTTGCTTCT
CCL1-F (M)	GCT TAC GGT CTC CAA TAG CTG C
CCL1-R (M)	GCT TTC TCT ACC TTT GTT CAG CC
CCL2-F (M)	TTAAAAACCTGGATCGGAACCAA
CCL2-R (M)	GCATTAGCTTCAGATTTACGGGT
CCl3-F (M)	ACT GCC TGC TGC TTC TCC TAC A
CCl3-R (M)	ATG ACA CCT GGC TGG GAG CAA A
CCl5-F (M)	CCT GCT GCT TTG CCT ACC TCT C
CCl5-R (M)	ACA CAC TTG GCG GTT CCT TCG A
CCl7-F (M)	CAG AAG GAT CAC CAG TAG TCG G
CCl7-R (M)	ATA GCC TCC TCG ACC CAC TTC T
CCR1-F (H)	GAC TAT GAC ACG ACC ACA GAG T
CCR1-R (H)	CCA ACC AGG CCA ATG ACA AAT A
CCL2 R (H)	TGG AAT CCT GAA CCC ACT TCT
CCL2 F (H)	CAG CCA GAT GCA ATC AAT GCC
CCL3 R (H)	CGG CTT CGC TTG GTT AGG AA
CCL3 F (H)	AGT TCT CTG CAT CAC TTG CTG
CCL5 R (H)	CTC TGG GTT GGC ACA CAC TT
CCL5 F (H)	CCA GCA GTC GTC TTT GTC AC
CCL7 R (H)	CAT TCC TTA GGC GTG ACC AT
CCL7 F (H)	TGA AAA CCC CAA CTC CAA AG
CCR2-F (M)	ATC CAC GGC ATA CTA TCA ACA TC
CCR2-R (M)	CAA GGC TCA CCA TCA TCG TAG
CD36-F (M)	ATGGGCTGTGATCGGAACTG
CD36-R(M)	GTCTTCCCAATAAGCATGTCTCC
CD74 F (M)	GCTGGATGAAGCAGTGGCTCTT
CD74 R (M)	GATGTGGCTGACTTCTTCCTGG
Cd209a-F (M)	GCACTCCATCAAAGGCTTTGGC
Cd209a-R (M)	CAAACAGCTAGGAAGAGCACCTG

Cd209b-F (M)	GGCTAAAGGACCAACCTGGATG
Cd209b-R (M)	CTCACCGATGTTGTTAGGCTCC
Cd209c-F (M)	GGAATGACTCTGTCAATGCCTGC
Cd209c-R (M)	GCCTTTCTCTTTAGAAGTCTGCTG
Cd209d-F (M)	ATTGGCACAACTCCACCACTGC
Cd209d-R (M)	ATCCAGGTTGGTCCTCTAGCCT
CD11b-F (M)	ATGGACGCTGATGGCAATACC
CD11b-R(M)	TCCCCATTCACGTCTCCCA
Ces1d F (M)	CAGAGGATGAACTACTGGAGACC
Ces1d R (M)	CTGGTGCCTTTGGCAGAACTAC
CXCL1-F(H)	AGC TTG CCT CAA TCC TGC ATC C
CXCL1-R(H)	TCC TTC AGG AAC AGC CAC CAG T
CXCL2-F(H)	GGC AGA AAG CTT GTC TCA ACC C
CXCL2-R(H)	CTC CTT CAG GAA CAG CCA CCA A
CXCL3-F(H)	TTC ACC TCA AGA ACA TCC AAA GTG
CXCL3-R(H)	TTC TTC CCA TTC TTG AGT GTG GC
CXCL10-F(H)	GGT GAG AAG AGA TGT CTG AAT CC
CXCL10-R(H)	GTC CAT CCT TGG AAG CAC TGC A
CX3CR1-F (M)	GAG TAT GAC GAT TCT GCT GAG G
CX3CR1-R (M)	CAG ACC GAA CGT GAA GAC GAG
CXCL1-F (M)	TCC AGA GCT TGA AGG TGT TGC C
CXCL1-R (M)	AAC CAA GGG AGC TTC AGG GTC A
CXCL2-F (M)	CAT CCA GAG CTT GAG TGT GAC G
CXCL2-R (M)	GGC TTC AGG GTC AAG GCA AAC T
CXCL5-F (M)	CCG CTG GCA TTT CTG TTG CTG T
CXCL5-R (M)	CAG GGA TCA CCT CCA AAT TAG CG
CXCL10-F (M)	ATC ATC CCT GCG AGC CTA TCC T
CXCL10-R (M)	GAC CTT TTT TGG CTA AAC GCT TTC
Dab2-F (M)	CTCTTCAAAGGCAATGCTCCTGC
Dab2-R (M)	TATGGCTCCTGGGACCACAGTT
Gata6-F (M)	TTG CTC CGG TAA CAG CAG TG
Gata6-R (M)	GTG GTC GCT TGT GTA GAA GGA
GSTM1-F (M)	TGTTTGAGCCCAAGTGCCTGGA
GSTM1-R (M)	TAGGTGTTGCGATGTAGCGGCT
GSTM2-F (M)	AGAGCAATGCCATCCTGCGCTA
GSTM2-R (M)	GTGTCCATAGCCTGGTTCTCCA
GSTM3-F (M)	AGAGCAATGCCATCCTGCGCTA
GSTM3-R (M)	GGTTCTCCAAAGTATCCACACGG
GSTM4-F (M)	ATCACGCAGAGCAATGCCATCC
GSTM4-R (M)	GGAGACATCCATAGCCTGGTTC
Gstp1-F (M)	TGGAAGGAGGAGGTGGTTACCA
Gstp1-R (M)	GGTAAAGGGTGAGGTCTCCATC
Gstp2-F (M)	TGGAAGGAGGAGGTGGTTACCA
Gstp2-R (M)	GGTAAAGGGTGAGGTCTCCATC
Gstt1-F (M)	TATCCCGTTCCAGATGCACACG
Gstt1-R (M)	CCAGGTAGAGCAAGATAGCCAC
Gstt2-F (M)	ATGCCGACAACATCCGTGGTAC
Gstt2-R (M)	AGCTGTTGCAGAACCAGGACCA
Gstt3-F (M)	CAGGTGCTAGAGGACAAGTTCC

Gstt3-R (M)	GATTTTGCAGCCAGCACTGACAG
Gata1-F (M)	TGGGGACCTCAGAACCCTTG
Gata1-R (M)	GGCTGCATTTGGGGAAGTG
Gata2-F (M)	CACCCCGCCGTATTGAATG
Gata2-R (M)	CCTGCGAGTCGAGATGGTTG
GSTM5 F (M)	GAAGCCAATGGCTGGACGTGAA
GSTM5 R (M)	CGTGCGATGTATCTCAGGATGG
Hmga2-F (M)	AGAGGAAGACCCAAAGGCAGCA
Hmga2-R (M)	GAGCAGGCTTCTTCTGAACGAC
ID2-F (M)	TCACCAGAGACCTGGACAGAAC
ID2-R (M)	TGCTATCATTCGACATAAGCTCAG
ID3-F (M)	GCGTGTCATAGACTACATCCTCG
ID3-R (M)	GTCCTTGGAGATCACAAGTTCC
IL1B-F (H)	TTC GAC ACA TGG GAT AAC GAG G
IL1B-R (H)	TTT TTG CTG TGA GTC CCG GAG
Il1b-F (M)	GCAACTGTTCCTGAACTCAACT
Il1b-R (M)	ATCTTTTGGGGTCCGTCAACT
IL6-F (M)	TAG TCC TTC CTA CCC CAA TTT CC
IL6-R (M)	TTG GTC CTT AGC CAC TCC TTC
IL-8-F (H)	CTT GGC AGC CTT CCT GAT TT
IL-8-R (H)	TTC TTT AGC ACT CCT TGG CAA AA
IL-10-F (H)	GGT TGC CAA GCC TTG TCT GA
IL-10-R (H)	AGG GAG TTC ACA TGC GCC T
IL10-F (M)	CCCATTCCTCGTCACGATCTC
IL10-R (M)	TCAGACTGGTTTGGGATAGGTTT
iNOS-F (M)	GTTCTCAGCCCAACAATACAAGA
iNOS-R (M)	GTGGACGGGTCGATGTCAC
Irs1-F (M)	TGTCACCCAGTGGTAGTTGCTC
Irs1-R (M)	CTCTCAACAGGAGGTTTGGCATG
Irs2-F (M)	CCAGTAAACGGAGGTGGCTACA
Irs2-R (M)	CCATAGACAGCTTGGAGCCACA
Irs3-F (M)	GCAAGCATGGTACAGCGCCCT
Irs3-R (M)	CCTTGGACCGCAGTGTCACAG
Lyve1-F (M)	ACCAGGTAGAGTCAGCGCAGAA
Lyve1-R (M)	CAGGACACCTTTGCCATTCTTCC
Ly6c1-F (M)	GCAGTGCTACGAGTGCTATGG
Ly6c1-R (M)	ACTGACGGGTCTTTAGTTTCCTT
Mest-F (M)	GTGGTGGGTCCAAGTAGGG
Mest-R (M)	AAGCACAACTATCTCAGGGCT
Mgst1-F (M)	TGCGACCGCATTCCAGAGGATA
Mgst1-R (M)	TCCACCTTCTCGTCAGTGCGAA
Msr1-F (M)	CGCACGTTCAATGACAGCATCC
Msr1-R (M)	GCAAACACAAGGAGGTAGAGAGC
Mt1-F (M)	ACCTCCTTGCAAGAAGAGCTGCT
Mt1-R (M)	GCTGGGTTGGTCCGATACTATT
Mt2-F (M)	CGTGGGCTGTGCGAAGTGCTC
Mt2-R (M)	AAAGGCTAGGCTTCTACATGGTC
Olr1-F (M)	GTCATCCTCTGCCTGGTGTTGT
Olr1-R (M)	TGCCTTCTGCTGGGCTAACATC

PDGFa-F (M)	GAGGAAGCCGAGATACCCC
PDGFa-R (M)	TGCTGTGGATCTGACTTCGAG
PDGFc-F (M)	GCCAAAGAACGGGGACTCG
PDGFc-R (M)	AGTGACAACTCTCTCATGCCG
SerpinB2-1F (H)	ACCATGGCCATGGTCTACAT
SerpinB2-1R (H)	ATCTGCTGCATGAACCCACA
SERPIN B2-F (M)	GCT CAA CAA TCA ACA CAC CAC
SERPIN B2-R (M)	GGT AGC AGG TTT GGG ATT TCA
Sfrp5 F (M)	GAGATGCTGCACTGCCACAAGT
Sfrp5 R (M)	TGCTCCATCTCACACTGGGCAC
Slc2a4-F (M)	GTGACTGGAACACTGGTCCTA
Slc2a4-R(M)	CCAGCCACGTTGCATTGTAG
SOD3-F (M)	GACCTGGTTGAGAAGATAGGCG
SOD3-R (M)	TGGCTGATGGTTGTACCCTGCA
Tgfb2-F (M)	CTTCGACGTGACAGACGCT
Tgfb2-R (M)	GCAGGGGCAGTGTAAACTTATT
Tgfbr2-F (M)	CCTACTCTGTCTGTGGATGACC
Tgfbr2-R (M)	GACATCCGTCTGCTTGAACGAC
TNFa-F (M)	CTTCTGTCTACTGAACTTCGGG
TNFb-R (M)	CAGGCTTGTCACTCGAATTTTG
TNF-F (H)	GAG GAC CTG GGA GTA GAT GAG
TNF-R (H)	CCT CTC TCT AAT CAG CCC TCT G

*M: Mouse, H: Human, F: Forward, R: Reverse

Supplementary Table 3: This table includes the primer sequences used for the quantitative PCR experiments.
