# Supplementary material for: Tissue-resident macrophage survival depends on mitochondrial function regulated by SerpinB2 in chronic inflammation
Source: Nat Commun. 2026 Feb 12;17:1493. doi: 10.1038/s41467-026-69196-4 (PMC12902017; doi:10.1038/s41467-026-69196-4)
Supplement: Supplementary file 2 — Description of Additional Supplementary Files [file 41467_2026_69196_MOESM2_ESM.pdf]

Description of Additional Supplementary File

Supplementary Video 1: VAT of lean mice contains higher numbers of resident macrophages than monocyte-derived macrophages. VAT macrophage subsets were enumerated in lean $CX_3CR1^{creER/+}$ $ROSA^{tdTomato}$ mice using intravital microscopy. VAT monocyte-derived macrophages ($tdTomato^+$) and resident macrophages ($tdTomato^-$) were detected.

Supplementary Video 2: VAT of obese mice contains fewer resident macrophages than monocyte-derived macrophages. VAT macrophage subsets were enumerated in obese $CX_3CR1^{creER/+}$ $ROSA^{tdTomato}$ mice using intravital microscopy. VAT monocyte-derived macrophages ($tdTomato^+$) and resident macrophages ($tdTomato^-$) were detected.
