# Supplementary material for: Author Correction: Tissue-resident macrophage survival depends on mitochondrial function regulated by SerpinB2 in chronic inflammation
Source: Nat Commun. 2026 Apr 17;17:3573. doi: 10.1038/s41467-026-72166-5 (PMC13090332; doi:10.1038/s41467-026-72166-5)
Supplement: Supplementary file 1 — Original, uncorrected Fig. 2 [file 41467_2026_72166_MOESM1_ESM.pdf]

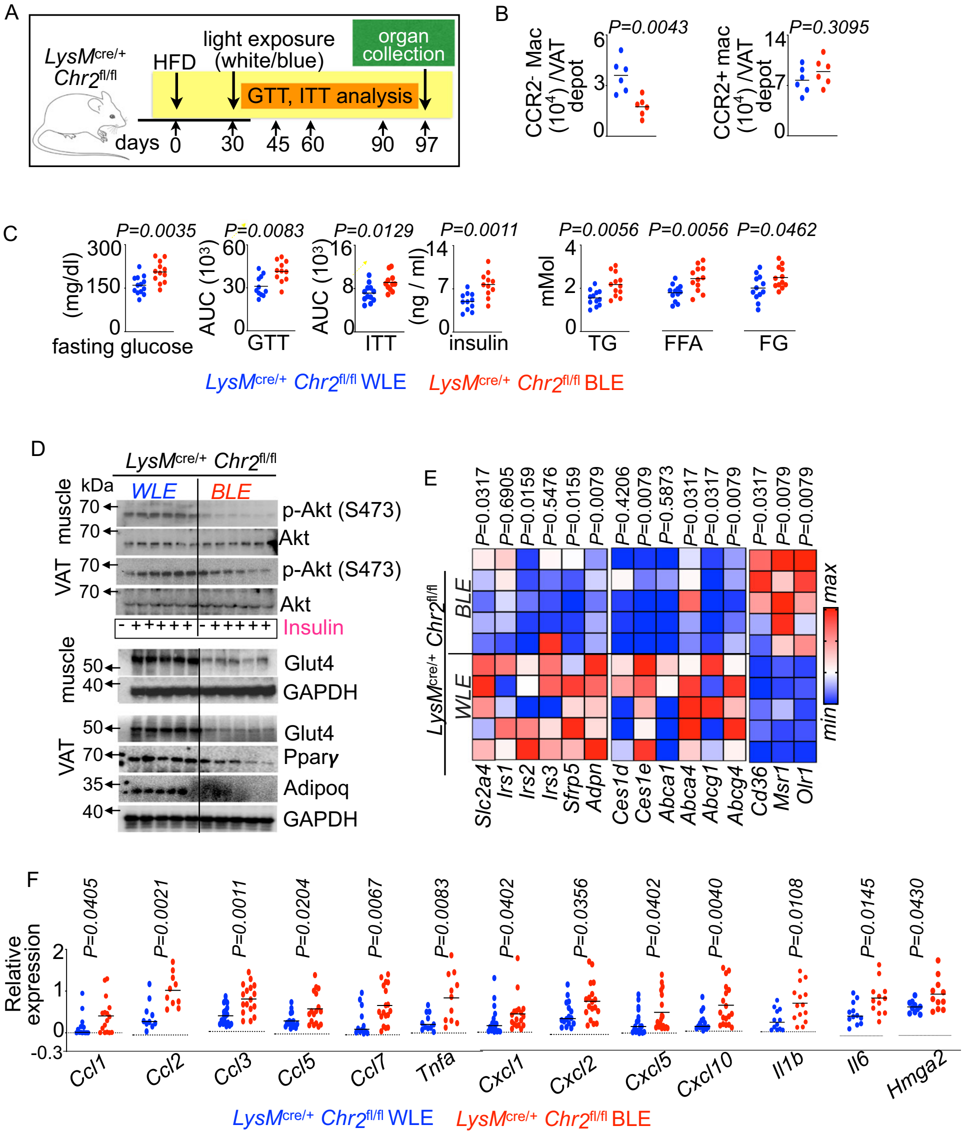


**Fig. 2 | VAT resident macrophage loss exacerbates obesity-induced metabolic complications. A**–**F** Blue/white light-exposed (BLE and WLE, respectively) *LysM*$^{cre/+}$ *Chr2*$^{fl/fl}$ mice were fed with an HFD. **A** Schematic diagram showing the experimental design. **B** Enumeration of the VAT macrophage subsets using flow cytometry (n = 6/group). **C** GTT and ITT were performed. The concentrations of fasting blood glucose, serum insulin, triglycerides (TG), free fatty acids (FFA), and free glycerol (FG) were evaluated (n = 13 for WLE and 12 for BLE, combined data of 2 independent experiments). **D** Immunoblot images of the represented proteins in muscle and VAT (n = 5–6/group). **E**, **F** qPCR quantification of the expression of the indicated metabolic and inflammatory genes measured in VAT represented by heat maps (**E**) (n = 5/group) and bar graphs (**F**) (n = 15/group). Mean ± s.e.m. * $P < 0.05$, ** $P < 0.01$. The Mann–Whitney test (two-tailed) was used to determine the significance between two groups.
